# Urethral Dysfunction in Female Mice with Estrogen Receptor β Deficiency

**DOI:** 10.1371/journal.pone.0109058

**Published:** 2014-10-02

**Authors:** Yung-Hsiang Chen, Chao-Jung Chen, Shuyuan Yeh, Yu-Ning Lin, Yang-Chang Wu, Wen-Tsong Hsieh, Bor-Tsang Wu, Wen-Lung Ma, Wen-Chi Chen, Chawnshang Chang, Huey-Yi Chen

**Affiliations:** 1 Graduate Institute of Integrated Medicine, College of Chinese Medicine, School of Pharmacy, College of Pharmacy, Department of Pharmacology, Department of Physical Therapy, Graduate Institute of Rehabilitation Science, China Medical University, Taichung, Taiwan; 2 Departments of Medical Research, Urology, and Obstetrics and Gynecology, Sex Hormone Research Center, China Medical University Hospital, Taichung, Taiwan; 3 Department of Urology, George H Whipple Laboratory for Cancer Research, Wilmot Cancer Center, University of Rochester Medical Center, Rochester, New York, United States of America; John Hopkins University School of Medicine, United States of America

## Abstract

Estrogen has various regulatory functions in the growth, development, and differentiation of the female urogenital system. This study investigated the roles of ERβ in stress urinary incontinence (SUI). Wild-type (ERβ^+/+^) and knockout (ERβ^−/−^) female mice were generated (aged 6–8 weeks, n = 6) and urethral function and protein expression were measured. Leak point pressures (LPP) and maximum urethral closure pressure (MUCP) were assessed in mice under urethane anesthesia. After the measurements, the urethras were removed for proteomic analysis using label-free quantitative proteomics by nano-liquid chromatography–mass spectrometry (LC-MS/MS) analysis. The interaction between these proteins was further analysed using MetaCore. Lastly, Western blot was used to confirm the candidate proteins. Compared with the ERβ^+/+^ group, the LPP and MUCP values of the ERβ^−/−^ group were significantly decreased. Additionally, we identified 85 differentially expressed proteins in the urethra of ERβ^−/−^ female mice; 57 proteins were up-regulated and 28 were down-regulated. The majority of the ERβ knockout-modified proteins were involved in cell-matrix adhesion, metabolism, immune response, signal transduction, nuclear receptor translational regelation, and muscle contraction and development. Western blot confirmed the up-regulation of myosin and collagen in urethra. By contrast, elastin was down-regulated in the ERβ^−/−^ mice. This study is the first study to estimate protein expression changes in urethras from ERβ^−/−^ female mice. These changes could be related to the molecular mechanism of ERβ in SUI.

## Introduction

Stress urinary incontinence (SUI) is defined as the involuntary leakage of urine under stress conditions such as coughing and sneezing [1]–[3]. The effects of birth trauma, menopause, and aging may contribute to the development of SUI [4]. Although improvement has been made in SUI treatment [5], our comprehension of the molecular mechanisms underlying this condition is inadequate.

Estrogen exerts a variety of regulatory functions on growth, development, and differentiation in the female urogenital system [6]. Estrogen actions are mediated by estrogen receptors (ERs) [7], encoded by two distinct genes, ERα and ERβ. Due to the female predominance of autoimmune diseases, the role of gender and sex hormones in the immune system is of interest. The primary effects of estrogen are mediated via ERs that are expressed on most immune cells. ERs are nuclear hormone receptors that can either directly bind to estrogen response elements in gene promoters or serve as cofactors with other transcription factors. ERs have prominent effects on immune function in both the innate and adaptive immune responses [8]. The discovery of ERβ in 1996 stimulated great interest in the physiological roles and molecular mechanisms of its action. ERβ plays a major role in mediating estrogen action in several tissues and organ systems, including the immune system [9]. Genetic deficiency of ERβ had minimal to no effect in autoimmune models [8].

ERβ-deficient mice have normal estrogen levels and skeletal axial growth is affected in adult female mice [10]. Skeletal muscle is also an estrogen-responsive tissue and there is a plausible mechanism of estrogenic action in skeletal muscle through ERs. It has been hypothesized that ovariectomy- and age-induced estradiol deficiency should result in ER changes in skeletal muscle and conversely, that estradiol replacement reverses these effects [11]. There are reports of age-related ERβ changes in different tissues, but the biological effects of ERβ deficiency in urethra and skeletal muscle are unclear [12]. In urogynecology, the efficacy of estrogen for SUI in postmenopausal women is still controversial [13]. Therefore, the specific roles of estrogen and ERβ in SUI remained elusive.

Because of the limited availability of human tissue for study, animal models are an important adjunct in improving our understanding of SUI [14]. Over the last decade, animal models of SUI have increasingly been used to understand the pathogenesis of SUI [15]. Vaginal distension (VD) [16] and pudendal nerve transaction [17] have been used for creation of SUI in rats, as evidenced by lowered leak point pressures (LPP) on urodynamic testing. The use of mice in various lines of translational research has made available transgenic and knockout technologies for conducting mechanistic studies of varied target diseases [18], [19]. The C57BL/6 mouse, for example, has been widely used for genetic manipulation in previous studies concerning urinary and pelvic disorders [20]. Interestingly, the decrease of ER in the pelvic floor tissues in pelvic organ prolapse (POP) patients may be closely related to the occurrence of SUI [4].

Proteomics approaches to identify and quantify the entire protein content (proteome) of a tissue at a given time may provide insights into the mechanisms of diseases [21]. Our aim was to understand the molecular mechanism of ERβ in SUI and in this study using label free quantitative proteomics by nanoLC-MS/MS (liquid chromatography–mass spectrometry) analysis we identified candidate target proteins in urethra from ERβ deficiency female mice.

## Results

### Decreased LPP and maximum urethral closure pressure (MUCP) in ERβ^−/−^ mice

**Figure 1:**
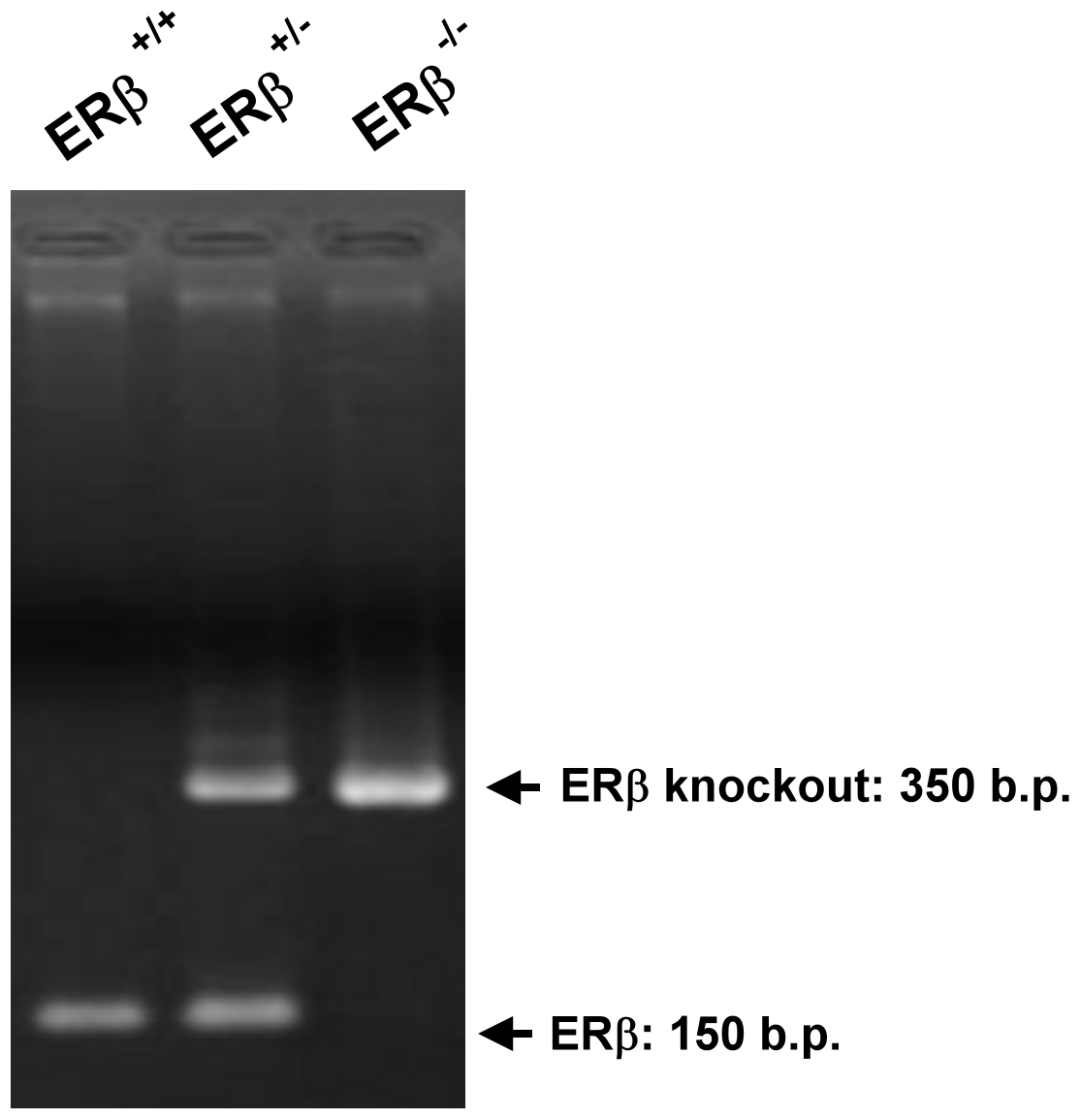
Genotyping of different ERβ mutant mice. Lane 1, ERβ^+/+^ mice; Lane 2, ERβ^+/-^ mice; Lane 3, ERβ^-/-^ mice.

ERβ genotyping was based on genomic sequence of ERβ exon 3. We used the primer sequence as below to identify PstI site insert on ERβ exon 3 transgene animal. The size of fragments for wild-type and knockout allele was about 150 and 350 b.p., respectively (**Figure 1**). Female C57BL/6 ERβ^+/+^ mice, aged 6–8 weeks, were used as control.

**Figure 2:**
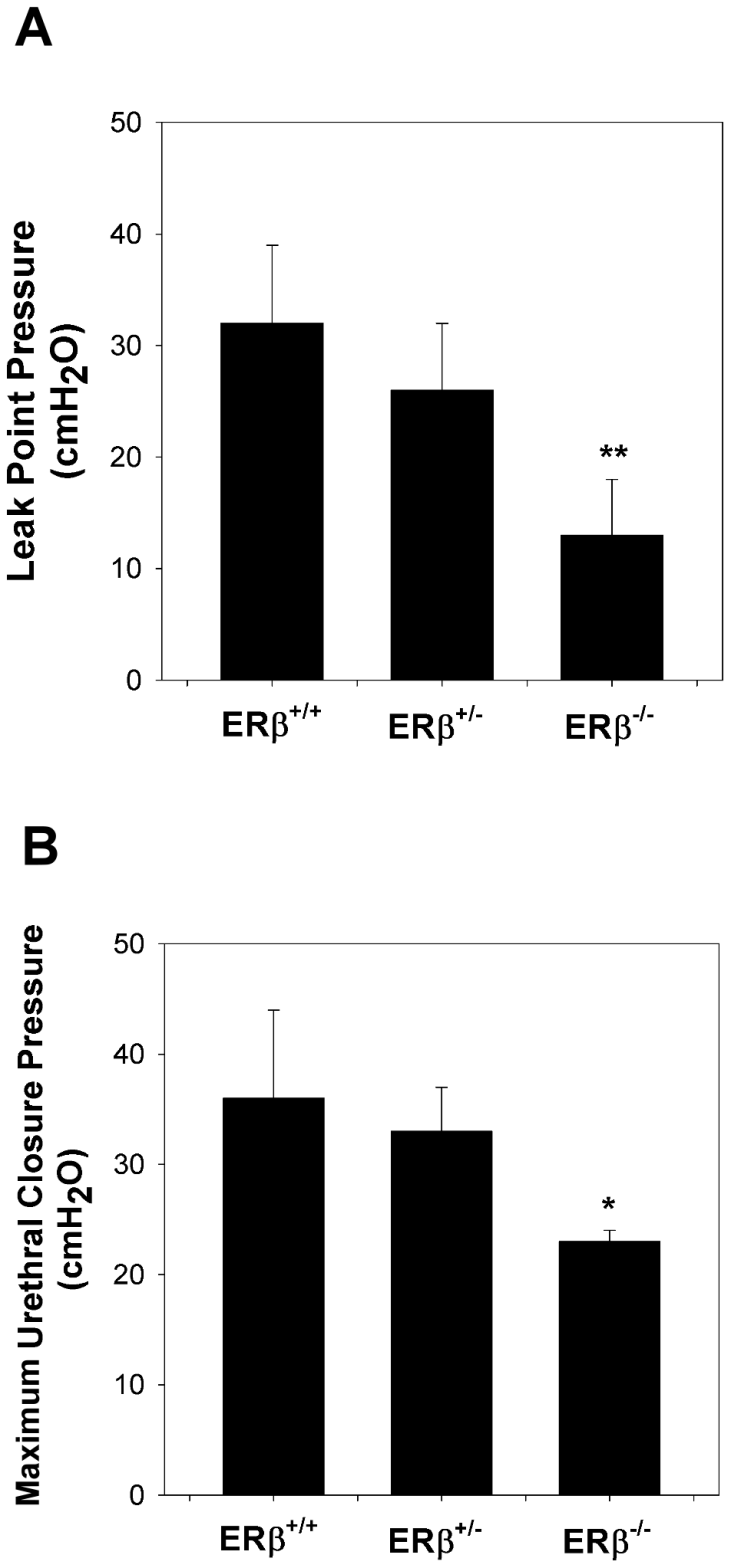
Decreased urodynamic testing in ERβ^−/−^ mice. (A) LPP and (B) MUCP values in the different groups. Each bar represents the mean ± standard deviation of six individual mice. *P<0.05 different from the value in the control group. **P<0.01 different from the value in the control group.

LPP and MUCP values were slightly decreased in the ERβ^+/-^ group without statistical significance. By contrast, LPP and MUCP values were significantly decreased in the ERβ^−/−^ group compared with the ERβ^+/+^ group (**Figures 2A and 2B**).

### Protein expression profile from proteomic analysis

**Figure 3:**
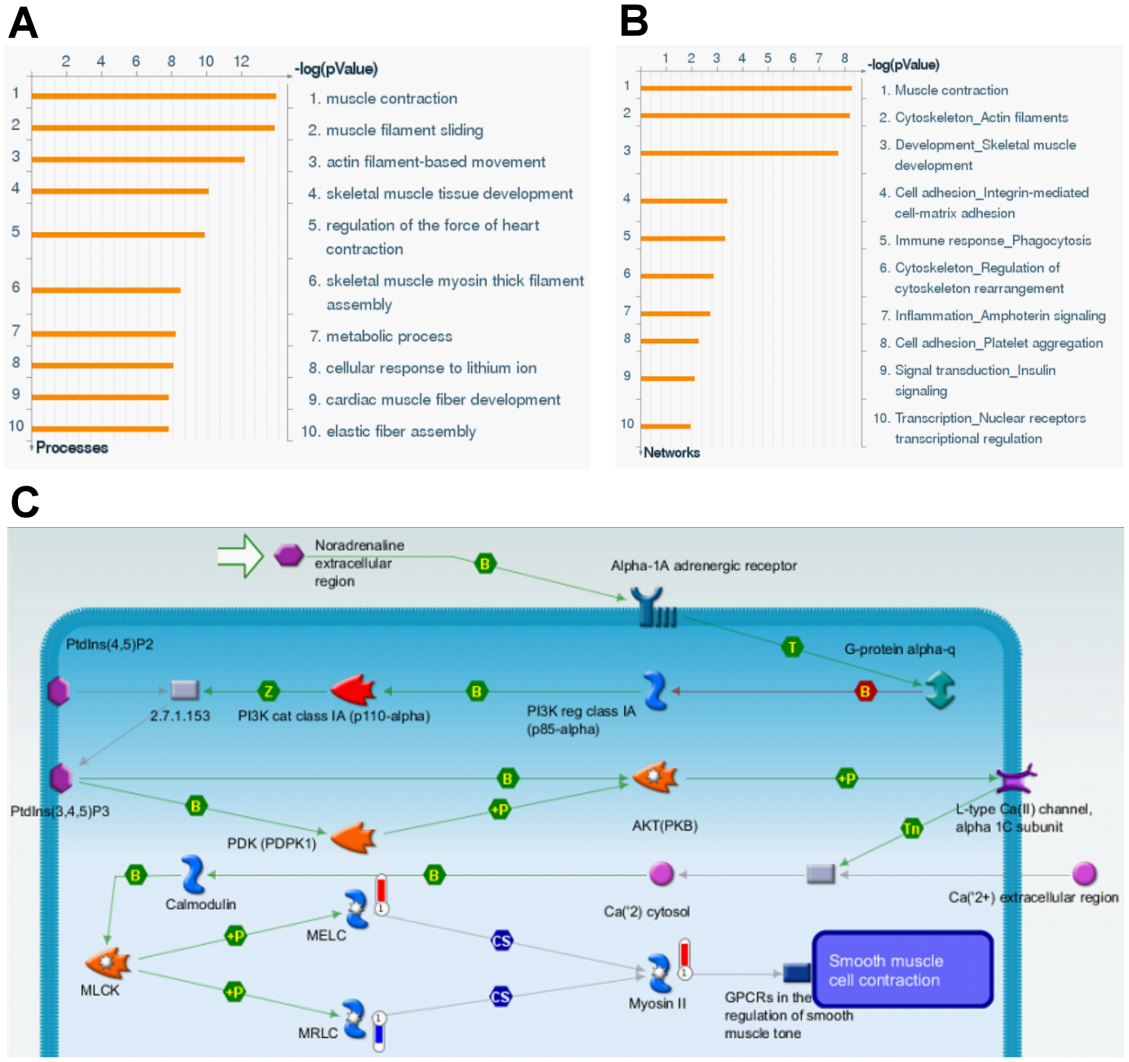
Protein expression profile from proteomic analysis. In term of (A) Gene Ontology and (B) biological networks databases, the differentially expressed proteins of urethra from ERβ^-/-^ female mice were divided into different categories. (C) Biological network analysis for differentially expressed proteins of urethra from ERβ^-/-^ female mice using MetaCore mapping tool. The network was generated using shortest path algorithm to map interaction between the proteins.

We identified 85 urethra proteins differentially expressed with statistical significance in ERβ^+/+^ and ERβ^−/−^ female mice. Additionally, 57 proteins were up-regulated (**Table 1**) and 28 were down-regulated (**Table 2**). The majority of the ERβ knockout-modified proteins were involved in cell-matrix adhesion, metabolism, immune response, signal transduction, nuclear receptor translational regelation, and muscle contraction and development (**Figures 3A, 3B, and 3C**).

**Table 1:** Up-regulated proteins in ERβ^-/-^ mouse urethra.

Accession	Protein	Ratio
A1AT1_MOUSE	α-1-antitrypsin 1-1	1.5
KAD1_MOUSE	Adenylate kinase isoenzyme 1	1.5
MYH1_MOUSE	Myosin-1	1.5
RAC3_MOUSE	Ras-related C3 botulinum toxin substrate 3	1.5
TRFE_MOUSE	Serotransferrin	1.5
B3AT_MOUSE	Band 3 anion transport protein	1.5
H3C_MOUSE	Histone H3.3C	1.5
RS9_MOUSE	40S ribosomal protein S9	1.6
GPX3_MOUSE	Glutathione peroxidase 3	1.6
GSTO1_MOUSE	Glutathione S-transferase Ω-1	1.6
A1AT2_MOUSE	α-1-antitrypsin 1–2	1.6
RD23B_MOUSE	UV excision repair protein RAD23 homolog B	1.6
FIBG_MOUSE	Fibrinogen γ chain	1.6
FBN1_MOUSE	Fibrillin-1	1.6
A1AT4_MOUSE	α-1-antitrypsin 1–4	1.6
RS14_MOUSE	40S ribosomal protein S14	1.6
DHB11_MOUSE	Estradiol 17-β-dehydrogenase 11	1.7
PEBP1_MOUSE	Phosphatidylethanolamine-binding protein 1	1.7
CASQ1_MOUSE	Calsequestrin-1	1.7
MYH4_MOUSE	Myosin-4	1.7
SPG16_MOUSE	Sperm-associated antigen 16 protein	1.7
K1C10_MOUSE	Keratin, type I cytoskeletal 10	1.7
PGS2_MOUSE	Decorin	1.7
KCRM_MOUSE	Creatine kinase M-type	1.7
MYP0_MOUSE	Myelin protein P0	1.7
WFS1_MOUSE	Wolframin	1.7
ITIH4_MOUSE	Inter α-trypsin inhibitor, heavy chain 4	1.7
HBB1_MOUSE	Hemoglobin subunit β-1	1.8
CO6A1_MOUSE	Collagen α-1(VI) chain	1.8
H14_MOUSE	Histone H1.4	1.8
APOE_MOUSE	Apolipoprotein E	1.8
LUM_MOUSE	Lumican	1.9
CLUS_MOUSE	Clusterin	1.9
CO6A2_MOUSE	Collagen α-2(VI) chain	1.9
MIME_MOUSE	Mimecan	1.9
HBA_MOUSE	Hemoglobin subunit α	1.9
MYH8_MOUSE	Myosin-8	2.0
PURA1_MOUSE	Adenylosuccinate synthetase isozyme 1	2.1
TNNT3_MOUSE	Troponin T, fast skeletal muscle	2.2
HMGB1_MOUSE	High mobility group protein B1	2.2
MPC2_MOUSE	Mitochondrial pyruvate carrier 2	2.3
MYG_MOUSE	Myoglobin	2.3
CAH2_MOUSE	Carbonic anhydrase 2	2.3
ILEUA_MOUSE	Leukocyte elastase inhibitor A	2.5
A1BG_MOUSE	α-1B-glycoprotein	2.6
MYL3_MOUSE	Myosin light chain 3	2.6
COPD_MOUSE	Coatomer subunit δ	3.0
IQGA1_MOUSE	Ras GTPase-activating-like protein IQGAP1	3.2
MK01_MOUSE	Mitogen-activated protein kinase 1	3.2
ASPN_MOUSE	Asporin	3.3
KV3A3_MOUSE	Ig κ chain V-III region MOPC 70	3.8
IGHM_MOUSE	Ig μ chain C region secreted form	4.2
NID1_MOUSE	Nidogen-1	4.3
ACDSB_MOUSE	Short/branched chain specific acyl-CoA dehydrogenase	4.5
IGKC_MOUSE	Ig κ chain C region	4.8
IGG2B_MOUSE	Ig γ-2B chain C region	5.3
GCAB_MOUSE	Ig γ-2A chain C region secreted form	13.4

**Table 2:** Down-regulated proteins in ERβ^-/-^ mouse urethra.

Accession	Protein	Ratio
HVM17_MOUSE	Ig heavy chain V region MOPC 47A	0.2
COEA1_MOUSE	Collagen α-1(XIV) chain	0.3
SERPH_MOUSE	Serpin H1	0.3
CAF17_MOUSE	Putative transferase CAF17 homolog, mitochondrial	0.4
AQP1_MOUSE	Aquaporin-1	0.4
NONO_MOUSE	Non-POU domain-containing octamer-binding protein	0.4
RS23_MOUSE	40S ribosomal protein S23	0.4
FABP4_MOUSE	Fatty acid-binding protein, adipocyte	0.4
ACLY_MOUSE	ATP-citrate synthase	0.4
GSTA4_MOUSE	Glutathione S-transferase A4	0.4
MYO1C_MOUSE	Unconventional myosin-Ic	0.4
NID2_MOUSE	Nidogen-2	0.5
DHX9_MOUSE	ATP-dependent RNA helicase A	0.5
CALR_MOUSE	Calreticulin	0.5
CF058_MOUSE	UPF0762 protein C6orf58 homolog	0.5
ATP5E_MOUSE	ATP synthase subunit epsilon, mitochondrial	0.5
RS3A_MOUSE	40S ribosomal protein S3a	0.5
MUG1_MOUSE	Murinoglobulin-1	0.5
PGAM1_MOUSE	Phosphoglycerate mutase 1	0.5
FAS_MOUSE	Fatty acid synthase	0.5
DHI1_MOUSE	Corticosteroid 11-beta-dehydrogenase isozyme 1	0.5
MYL9_MOUSE	Myosin regulatory light polypeptide 9	0.5
LEG3_MOUSE	Galectin-3	0.5
GSTM1_MOUSE	Glutathione S-transferase Mu 1	0.5
COX2_MOUSE	Cytochrome c oxidase subunit 2	0.5
CPNE3_MOUSE	Copine-3	0.5
SPA3K_MOUSE	Serine protease inhibitor A3K	0.5
PPIB_MOUSE	Peptidyl-prolyl cis-trans isomerase B	0.5

### Myosin, collagen, and elastin expressions in the urethra of ERβ^−/−^ mice

We further focused on urethral dysfunction-related proteins including myosin, collagen, and elastin and confirmed their expressions by Western blot analysis. There is a contradiction between the different subtypes of urethral dysfunction-related protein expressions. For example, four types of myosin (myosin-1, 4, 8, and myosin light chain 3) that were overexpressed in ERβ^−/−^ female mice, whereas other two types of myosin (unconventional myosin-Ic and myosin regulatory light polypeptide 9) were decreased. Thus, the common commercial available antibodies, including anti-myosin heavy chain (clone A4.1025), anti-collagen α-1(III) (FH-7A), and anti-elastin (BA-4), were chosen for the subsequent Western blot analysis.

**Figure 4:**
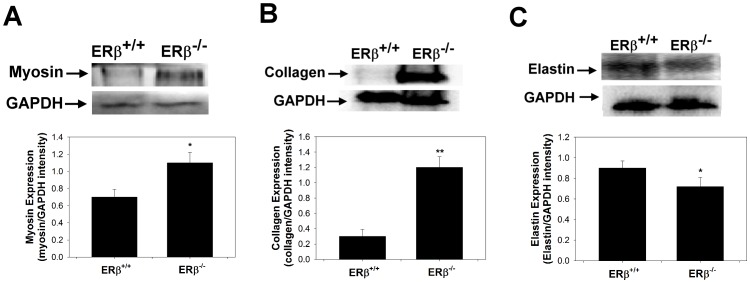
Urethral dysfunction-related proteins expressions. Alterations of (A) myosin, (B) collagen, and (C) elastin expressions in urethra as indicated by Western blot analyses. The values are calculated by intensity of each band (ratio of target protein/GAPDH) and expressed as mean ± standard deviation of six individual mice. *Significantly different from the value in the control group (*P*<0.05), ** *P*<0.01.

Myosin (**Figure 4A**) and collagen (**Figure 4B**) expressions in the urethra was significantly increased in the ERβ^−/−^ group as compared with the ERβ^+/+^ group. By contrast, elastin (**Figure 4C**) expression in the urethra was significantly decreased in the ERβ^−/−^ group.

## Discussion

Estrogen actions mediated by ERs are known to modulate lower urinary tract (LUT) trophicity [22]. In the present study, LPP and MUCP values were significantly decreased in the ERβ^−/−^ group compared with the ERβ^+/+^ group, indicating an important role of ERβ in SUI.

To the best of our knowledge, this is the first study that estimates the changes in protein expression related to ERβ in SUI. We used nanoLC-MS/MS analysis to evaluate the proteomic profile of urethral samples collected from ERβ^+/+^ and ERβ^−/−^ mice. We found 85 differentially expressed proteins between ERβ^+/+^ and ERβ^−/−^ female mice. The majority of the identified ERβ knockout-modified proteins were involved in cell-matrix adhesion, metabolism, immune response, signal transduction, nuclear receptor translational regulation, and muscle contraction and development. We further focused on urethral dysfunction-related proteins including myosin, collagen, and elastin and confirmed their expressions by Western blot analyses.

Four of the proteins that exhibited overexpression in ERβ^−/−^ female mice were related to myosin; specifically, Myosin-1, 4, 8, and myosin light chain 3 were overexpressed in ERβ^−/−^ female mice, whereas other two types of myosin (unconventional myosin-Ic and myosin regulatory light polypeptide 9) were decreased. Myosin proteins are composed of both heavy and light chains and are essential components of muscles. Myosin heavy chains help to determine the speed of muscle contraction; in contrast, the role of myosin regulatory light polypeptide 9 (whose expression was 50% decreased in ERβ^−/−^ female mice) is unclear. Since myosin is responsible for muscle contraction, this overexpression of myosin heavy chains may be a mechanism to counteract the loss of normal urethral function in SUI. This finding is consistent with previous works showing overexpression myosin genes in the pubococcygeus muscle of women with POP [23], [24].

The phenotype of the ERβ^−/−^ mice with respect to collagen biosynthesis appears to be more complex. Collagen biosynthesis and deposition is a multiphase process, which is tightly regulated to maintain proper tissue homeostasis. Collagen production is regulated by a variety of molecules, including growth factors, cytokines, and hormones. However, the factors and pathways involved in this process are not fully defined [25]. In the present study, type III collagen (a fibrillar collagen that is found in extensible connective tissues such as skin, lung, and the vascular system, frequently in association with type I collagen) expression in the urethra was significantly increased in ERβ^−/−^ group as compared with that in the ERβ^+/+^ group, indicating an increased synthesis of collagen or a decreased proteolysis in the urethra. Several factors may have contributed to the high deposition of extracellular matrix (ECM) in ERβ^−/−^ mice, including elevated collagen synthesis by fibroblasts [26], [27], and a decreased expression of matrix metalloproteinases in mouse tissue. For example, ER retained their responsiveness to estradiol with respect to collagen biosynthesis. There is evidence that ERβ knockout plays a role in the development of SUI. In addition, ERβ deletion in mice has been described to lead to fibrosis in various tissues [28]. For example, Pedram *et al*. showed that in the hearts of ovariectomized female mice, cardiac hypertrophy and fibrosis were prevented by estradiol administration to wild type but not ERβ knockout rodents. Their results established the cardiac fibroblast as an important target for hypertrophic/fibrosis-inducing peptides the actions of which were mitigated by estrogen/ERβ acting in these stromal cells [29]. This supports the findings that collagen increases in the urethra following deletion of ERβ.

Certain alterations in connective tissue metabolism are known to be modified in postmenopausal women with genuine stress incontinence. Jackson *et al*. showed that treatment with oestrogen has profound effects upon pelvic collagen metabolism, stimulating collagen degradation via increased proteinase activity. While aged collagen is being lost, new collagen is synthesized as witnessed by the increase in the immature cross-links and the decrease in both mature cross-links and advanced glycation end-products. In the present study, collagen deposition contradicts previous reports; perhaps aged collagen degradation is merely an early response to estrogen stimulation [30]. Further studies within this field are indeed needed.

By contrast, elastin was down-regulated in ERβ^−/−^ mice. Our previous study [31] suggested that some ECM remodelling enzymes, including lysyl oxidase, are required for the oxidative deamination of amino acid residues in collagen and elastin molecules a step that is required for fibre cross-linking and are therefore essential for the stabilization of collagen fibrils and for the integrity and elasticity of mature elastin [32]. The assembly and cross-linking of elastin/collagen fibres is crucial for the recovery of tissue elasticity and urethral support. The loss of elasticity and resiliency might be attributed to an imbalanced cross-linking of collagen to elastin. When these urethral characteristics manifest in a severe form, descriptive terms such as low-pressure urethra, lead-pipe urethra (urethra and bladder neck areas are open at rest) [33], [34], or patulous urethra have been used. Because the bladder and urethra are composed of both active (smooth muscle) and passive elements (collagen and elastin) [35], [36], our study model characterizes a long-term phase of ERβ knockout, representing lower urinary dysfunction in menopause-related development of SUI.

Our study has certain limitations. First, the pelvic floor structure of the mouse, which is a quadruped and has a lax abdominal wall, is different from that of a human female; therefore, the results of this study need to be carefully applied to human subjects. Second, urodynamic studies were conducted under anaesthesia; fortunately, none of our subjects manifested any evidence of bladder instability, implying that detrusor overactivity was not present, and giving credence to our interpretation of fluid expulsion in the absence of increased bladder pressure as evidence of SUI. Third, since a fairly large number of the protein expression profiles were found from the proteomic study, it is difficult to confirm all the expression levels of potential target proteins by Western blot analysis. Fourth, the experimental conditions and the sample preparation technique could have affected the detection of some proteins. In label-free analysis (as in any other proteomics method), not all proteins can be resolved simultaneously; this is due to differences in their properties (such as hydrophobicity, charge, and solubility properties). The specific protocol used in our study is optimal for the solubility of cytosolic proteins [24]. Changes in the protein extraction protocol are needed for easier detection of ECM proteins.

In conclusion, our results suggest a role for ERβ in SUI. This pilot study is the first one to estimate protein expression changes in urethras from ERβ^+/+^ and ERβ^−/−^ female mice. These changes could be related to the molecular mechanism of ERβ in SUI. A further study confirming the expression levels of potential target proteins in urethras from ERβ^+/+^ and ERβ^−/−^ female mice with Western blot analysis and changing the protein extraction protocol for proteomic analysis is needed. These findings may also have therapeutic implications [37] and perhaps provide pharmacological strategy for the treatment of urethral dysfunction with ERβ agonists [38], [39].

## Materials and Methods

### Ethics statement

All animals were housed and handled in accordance with criteria outlined in the National Institutes of Health “Guide for Care and Use of Laboratory Animals”. The study was approved by the Institutional Animal Care and Use Committee of China Medical University (Reference number: 101-201-N). All efforts were made to ameliorate animal suffering. Animal sacrifice was performed by CO_2_ asphyxiation followed by cervical dislocation.

### Animals

ERβ knockout mice were generated as previously described [40], [41]. ERβ genotyping was based on genomic sequence of ERβ exon 3. We used the primer sequence as below to identify PstI site insert on ERβ exon 3 transgene animal. 5′-exon 3 (3145): GTT GTG CCA GCC CTG TTA CT (AY028415), 3′-exon 3 (8140): GGG CCA GCT CAT TCC ACT C 8161 (X76683). The size of fragments for wild-type and knockout allele was about 150 and 350 b.p., respectively. Female C57BL/6 ERβ^+/+^ mice, aged 6–8 weeks, were used as control (n = 6).

The mice underwent suprapubic bladder tubing (SPT) placement [31], [42]. LPP and MUCP were assessed in these mice under urethane (1 g/kg, i.p.) anesthesia. After measurements, the animals were sacrificed, and the urethras were removed for proteomic and further analyses.

### Suprapubic tube implantation

The surgical procedure was carried out under 1.5% isoflurane anesthesia according to previous methods [31], [42]. An SPT (PE-10 tubing, Clay Adams, Parsippany, NJ) was implanted in the bladder. Key points of the operation were as follows: (1) a midline longitudinal abdominal incision was made, 0.5 cm above the urethral meatus; (2) a small incision was made in the bladder wall, and PE-10 tubing with a flared tip was implanted in the bladder dome; and (3) a purse-string suture with 8-0 silk was tightened around the catheter, which was tunneled subcutaneously to the neck, where it exited the skin.

### LPP measurement

Two days after implanting the bladder catheter, the LPP was assessed in these mice under urethane anesthesia. The bladder catheter was connected to both a syringe pump and a pressure transducer. Pressure and force transducer signals were amplified and digitized for computer data collection at 10 samples per second (PowerLabs, AD Instruments, Bella Vista, Australia). The mice were placed supine at the level of zero pressure while bladders were filled with room temperature saline at 1 ml/h through the bladder catheter. If a mouse voided, the bladder was emptied manually using Crede's maneuver. The average bladder capacity of each mouse was determined after 3–5 voiding cycles. Subsequently, the LPP was measured in the following manner [31], [42]. When half-bladder capacity was reached, gentle pressure with one finger was applied to the mouse's abdomen. Pressure was gently increased until urine leaked, at which time the externally applied pressure was quickly removed. The peak bladder pressure was taken as the LPP. At least three LPPs were obtained for each animal, and the mean LPP was calculated [43], [44].

### Urethral pressure profile

Urethral pressure profile (UPP) was assessed in these mice under urethane (1 g/kg, i.p.) anesthesia. The bladder catheter (PE-10 tubing, Clay Adams, Parsippany, NJ) was connected to a syringe pump with room temperature saline at 1 ml/hr. The urethral catheter (PE-10 tubing, Clay Adams, Parsippany, NJ) was connected to a pressure transducer. A withdrawal speed of 10 µm per minute was used. Pressure and force transducer signals were amplified and digitized for computer data collection at 10 samples per second (PowerLabs, ADInstruments, Bella Vista, Australia). Three successive profiles were obtained in the supine position. The urethral closure pressure (Pclose) is the difference between the urethral pressure (Pure) and the bladder pressure (Pves): Pclose  =  Pure – Pves [45]. Maximum urethral pressure and MUCP were determined from the UPP measurements taken. The mice were sacrificed immediately after completing the measurements of LPP and MUCP, and the urethras were harvested [31], [42].

### Protein preparation

Frozen pieces of urethra were weighed and then pulverized with a liquid nitrogen-chilled mortar and pestle. Tissue powder was then homogenized in buffer (16 mmol/l potassium phosphate, pH 7.8, 0.12 mol/l NaCl, 1 mmol/l ethylenediaminetetraacetic acid) containing a protease inhibitor cocktail (Complete Mini, product number 11836153001, Roche Diagnostics, Penzberg, Germany), and then centrifuged at 10,000×*g*. The supernatant was removed, and the previous homogenization step was repeated after re-suspending the remaining tissue pellet in basic buffer. After removal of the second supernatant, the remaining tissue pellet was suspended in urea buffer (6.0 mol/l in above buffer), homogenized, and placed on a rotating rack for overnight extraction at 4°C. Thereafter, the samples were centrifuged (13,000 g for 30 min), and the supernatant was removed. Protein concentrations were determined using a bicinchoninic acid protein assay (Pierce, Rockford, IL) and standard curves of BSA in appropriate buffers. The proteomic study was further analyzed with label free proteomics.

### Label free quantitative proteomics by nanoLC-MS/MS analysis

The nanoLC-MS/MS was performed with a nanoflow UPLC system (UltiMate 3000 RSLCnano system, Dionex, Amsterdam, Netherlands) coupled with a captive spray ion source and hybrid Q-TOF mass spectrometer (maXis impact, Bruker). The sample was injected into a tunnel-frit trap column (C18, 5 µm, 100 Å, packed length of 2 cm, 375 µm od×180 µm id) with a flow rate of 8 µl/min and a duration of 5 min. The trapped analyses were separated by a commercial analytical column (Acclaim PepMap C18, 2 µm 100 Å, 75 µm×250 mm, Thermo Scientific, USA) with a flow rate of 300 nl/min. An acetonitrile/water gradient of 1%–40% within 90 min was used for peptide separation. For MS/MS detection, peptides with charge 2+, 3+ or 4+ and the intensity greater than 20 counts were selected for data dependent acquisition, which was set to one full MS scan (400–2000 m/z) with 1 Hz and switched to ten product ion scans (100–2000 m/z) with ten Hz.

The LC-MS/MS spectra were deisotoped, centroided, and converted to xml files using DataAnalysis (version 4.1, Bruker). The xml files were searched against the Swissport (release 51.0) database using the MASCOT search algorithm (version 2.2.07). The search parameters for MASCOT for peptide and MS/MS mass tolerance were 50 ppm and 0.07 Da, respectively. Search parameters were selected as Taxonomy – mus; enzyme–trypsin; fixed modifications – carbamidomethyl (C); variable modifications – oxidation (M). Peptides were considered as identified if their MASCOT individual ion score was higher than 25 (*P*<0.01).

Label free quantitative proteomics was achieved by LC-MS replicated runs (n = 4) of different groups. After LC-MS runs finished, LC-MS/MS runs of each group was performed for protein identification. LC-MS results were processed to have molecular features with DataAnalysis 4.1 (Bruker Daltonics, Germany), which were then loaded into ProfileAnalysis software 2.0 (Bruker Daltonics) for *t*-test comparison between two groups. The *t*-test results among different groups were further transferred to ProteinScape 3.0 (Bruker Daltonics) and combined with protein identification results of each group for the integration of quantified peptide information into each protein [46].

### Networks analysis using MetaCore

MetaCore (GeneGo, St. Joseph, MI) was used to map the differentially expressed proteins into biological networks. It is an integrated software suited for functional analysis of protein–protein, protein–DNA and protein compound interactions, metabolic and signaling pathways, and the effects of bioactive molecules [47]. Differentially expressed proteins were converted into gene symbols and uploaded into MetaCore for analysis. The biological process enrichment was analyzed based on Gene Ontology processes. For network analysis, three algorithms were used: (1) the direct interaction algorithm to map direct protein-protein interactions; (2) the shortest path algorithm to map shortest path for interaction between differentially expressed proteins; and (3) the analyze network algorithm to deduce top scoring processes that are regulated by differentially expressed proteins [42].

### Western blot analysis

Urethral tissue were prepared by homogenization of cells in a lysis buffer containing 1% IGEPAL CA-630, 0.5% sodium deoxycholate, 0.1% sodium dodecyl sulfate, aprotinin (10 mg/mL), leupeptin (10 mg/mL), and phosphate-buffered saline (PBS). Cell lysates containing 100 µg of protein were subjected to sodium dodecyl sulfate polyacrylamide gel electrophoresis and then transferred to a polyvinylidene fluoride membrane (Millipore Corp, Bedford, MA, USA). The membrane was stained with Ponceau S to verify the integrity of the transferred proteins and to monitor the unbiased transfer of all protein samples [48], [49]. Detection of myosin and collagen on the membranes was performed with an electrochemiluminescence kit (Amersham Life Sciences Inc, Arlington Heights, IL, USA) with the use of the antibody derived from rabbit (anti-myosin heavy chain (clone A4.1025), 1∶500 dilution, Millipore, MA, USA; anti-collagen α-1(III) (FH-7A) antibody, 1∶500 dilution, Abcam, Cambridge, UK; anti-elastin (BA-4) antibody, 1∶500 dilution, Abcam, Cambridge, UK). The intensity of each band was quantified using a densitometer (Molecular Dynamics, Sunnyvale, CA, USA) [50], [51].

### Statistical analyses

The changes of the target expressions were compared by Student's *t*-test or analysis of variance (ANOVA). One-way ANOVA and *post*-hoc test (Bonferroni correction) were given for more than two groups are being compared [52]. *P*-value less than 0.05 was considered statistically significant. All calculations were performed using the Statistical Package for Social Sciences (SPSS for Windows, SPSS Inc, Chicago, IL, USA).
